# Influence of hand dominance, gender, and body mass index on hand grip strength

**DOI:** 10.4102/sajp.v79i1.1923

**Published:** 2023-10-27

**Authors:** Mercy A. Agtuahene, Jonathan Quartey, Samuel Kwakye

**Affiliations:** 1 Department of Physiotherapy, Korle-Bu Teaching Hospital, Accra, Ghana; 2 Department of Physiotherapy, University of Ghana, Accra, Ghana; 3 Department of Physiotherapy, West Africa Football Academy, Sogakope, Ghana

**Keywords:** hand grip strength, hand dominance, body mass index, dynamometer, non-dominant hand

## Abstract

**Background:**

Hand grip strength (HGS) measurements serve as an objective measure of upper extremity function. Reliable hand strength evaluation is vital for assessing treatment effectiveness.

**Objectives:**

To determine the influence of hand dominance, gender, and body mass index (BMI) on HGS among university students in Ghana.

**Method:**

In our cross-sectional study of 304 participants, height, weight, and BMI were measured using a stadiometer and weighing scale. Hand grip strength was assessed with a dynamometer. We compared HGS in dominant and non-dominant hands for males and females using a paired *t*-test and analysed the correlation between grip strength and weight, height, and BMI using Pearson’s correlation coefficient.

**Results:**

The mean HGS for right-hand dominant (RHD) male participants was 35.62 kg (± 7.36) for the right hand compared with 32.84 kg (± 7.36) for the left hand. For females RHD the mean HGS in the right hand was 24.60 kg (± 6.42) compared to 22.12 kg (± 5.37) in the left hand. The mean weight, height and BMI of participants were 62.86 kg (± 10.30), 1.67 m (± 0.09) and 22.9 kg/m^2^ (± 4.9), respectively. A significant relationship existed between HGS and height (*r* = 0.492; *p* < 0.01) as well as HGS and BMI (*r* = 0.290; *p* < 0.01). However, no notable connection was found between HGS and weight (*r* = 0.001; *p* = 0.982).

**Conclusion:**

Hand grip strength was significantly stronger in the dominant hand of both males and females.

**Clinical implications:**

Physiotherapists should test HGS objectively and quantitatively for use in disease evaluation, diagnosis, and therapy.

## Introduction

Grip strength is a general term that refers to the muscular power and force that is generated by the forearm muscles denoting the maximum force or tension produced (Vaidya & Nariya 2021). Hand grip strength (HGS) can be measured by manual testing and dynamometry (Saranya, Poonguzhali & Karunakaran 2020). Dynamometer measurements are characterised by their higher sensitivity to change in comparison to manual muscle testing. The dynamometer measurement yields outcomes on a continuous scale, signifying that they offer a spectrum of values instead of distinct categories (Saranya et al. 2020). This continuum permits a more intricate evaluation of strength variations over time. Normative values play a pivotal role in grip strength assessment, representing average or standard measurements established within specific populations or reference groups. For instance, HGS ranged between 32 and 51 for males and 19 and 32 for females among student population in Asia (Pratt et al. 2021; McQuiddy et al. 2015). Our pioneering study represents the first of its kind in Ghana and holds the potential to define context-specific benchmarks for HGS. These benchmarks are of importance in fine-tuning physiotherapy approaches, formulating achievable targets for hand rehabilitation, and customising treatments for distinct demographics.

Hand grip strength is a physiological variable that is affected by several factors including age, gender and body size (Koley, Ghandi & Singh 2008). According to Zaccagni et al. (2020) HGS can also be influenced by age, laterality, practice in different physical activities, size of the grip, height, and body mass. Studies in Egypt (Mahmoud et al. 2020) and India (Rajesh et al. 2022) have reported the existence of strong correlations between grip strength and various anthropometric traits such as weight, height, and hand length. For instance, Rajesh et al. (2022) reported that hand dominance significantly impacted grip strength, with the dominant hand showing higher strength than the non-dominant hand, while body mass index (BMI) had no significant effect. Rajesh et al. (2022) also reported that weight had the strongest correlation with HGS in females, followed by BMI, while in males, hand span exhibited the strongest correlation with HGS, followed by weight and height.

Hand grip strength, objectively assessed with a dynamometer, holds predictive value for a range of distinct outcomes across various subjects such as cardiovascular health in elderly individuals, functional capacity in postoperative patients, and muscular development in athletes (Manini et al. 2022). While studies may not exhibit absolute consistency, they generally favour grip strength as a predictor of postoperative complications, cardiovascular mortality, and functional decline (Manini et al. 2022; Prasitsiriphon & Pothisiri 2018). This highlights the need for broader utilisation of grip strength as a screening tool. Hand dynamometers have consistently exhibited high validity and reliability in measuring HGS, with test-retest reliability ranging from 0.96 to 0.98 (Nikodelis et al. 2021; Reis & Arantes 2011). To evaluate the effectiveness of various treatment modalities or the outcomes of various procedures, a valid and reliable assessment of hand strength is therefore crucial as indicated by Bobos et al. (2020) and further indicates that measurements of grip strengths yield an objective indicator of the upper extremity’s functional integrity. Thomas et al. (2021) also establish that the most used technique for evaluating upper extremity muscular strength is handgrip measurements. Hand grip strength has been successfully applied to predict post-operative complications (Matthews et al. 2021), and has a direct relationship with nutritional status (Narendra et al. 2020).

Anecdotal observations suggest that subjective assessments dominate the evaluation of HGS in most physiotherapy departments across Ghana. However, it is noteworthy that comprehensive research specifically focused on HGS in Ghana remains scarce. While numerous studies have been conducted in other parts of the world, the unique population characteristics, cultural factors, and regional variations in Ghana may significantly influence HGS and its implications for physiotherapy interventions. Therefore, a study on HGS in Ghana is useful to help address this research gap and gain a better understanding of the population-specific factors affecting hand rehabilitation outcomes. However, it is important to acknowledge that certain hand injuries and conditions, such as tendon injuries and hand fractures, may require caution when applying HGS assessments during the initial stages of healing, as premature assessment could potentially disrupt the healing process and compromise patient safety. By objectively assessing the influence of hand dominance, gender, and BMI on HGS in healthy individuals in Accra, Ghana, our study provides valuable insights for optimising physiotherapy interventions and setting realistic goals in hand rehabilitation. Moreover, our findings may contribute to the existing body of knowledge on HGS and possibly serve as a foundation for future research and clinical practice in Ghana.

## Methods

Our cross-sectional study was conducted among undergraduate students of the College of Health Sciences who did not have any obvious hand injuries at the time of our study, and were recruited using a convenience sampling technique. Prior to their participation, all participants were provided with comprehensive information about the research objectives, procedures, potential risks, and benefits. Written informed consent was obtained from each participant, ensuring their voluntary agreement to take part in our study and their understanding that they could withdraw at any point without consequence. Students who could not move through full range in their shoulder, elbow, wrist, or finger joints, as well as those with deformities or disabilities in their upper limbs were excluded. The sample size was calculated using Taro Yamane’s (1967) formula:

$$n=N/(1+Ne^{2}),$$ [Eqn 1]

where *n* = minimum sample size, *N* = population size (1200), and *e* = margin of error (5%). Thus, the minimum sample size was 300.

### Data collection tool

**FIGURE 1:**
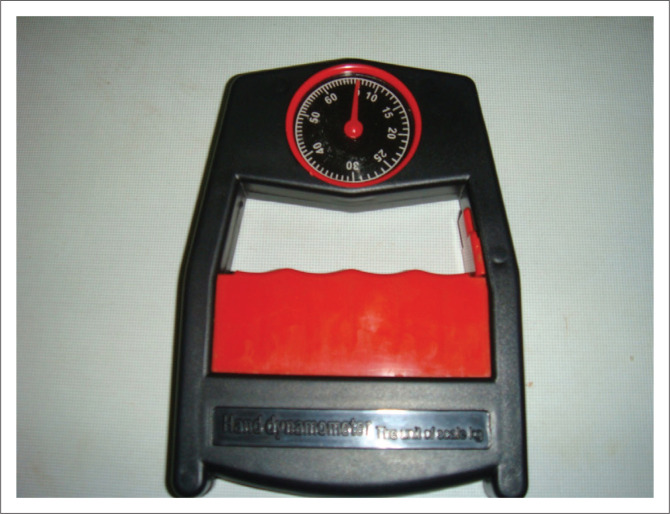
Haoyue hand dynamometer.

An analogue weighing scale (CAMBRY BR 3012, United Kingdom [UK]), stadiometer (Cescorf), and standard Haoyue hand dynamometer (Figure 1) were used to obtain weight, height and grip strength, respectively. The height and weight were measured according to Baharudin et al. (2017) and Kumar et al. (2014), methods for measuring, respectively. The analogue weighing scale and height meter used have been shown to have high validity and reliability with an interclass correlation (ICC) between 0.94 and 0.7 (Baharudin et al. 2017; Kumar et al. 2014). The procedure for measuring grip strength was performed as described by Andrade Fernandes et al. (2011). The hand dynamometer has been shown to be valid and reliable in measuring HGS with a systematic bias between 0.02 kg and 0.26 kg (España-Romero et al. 2010).

### Data collection procedure

**FIGURE 2:**
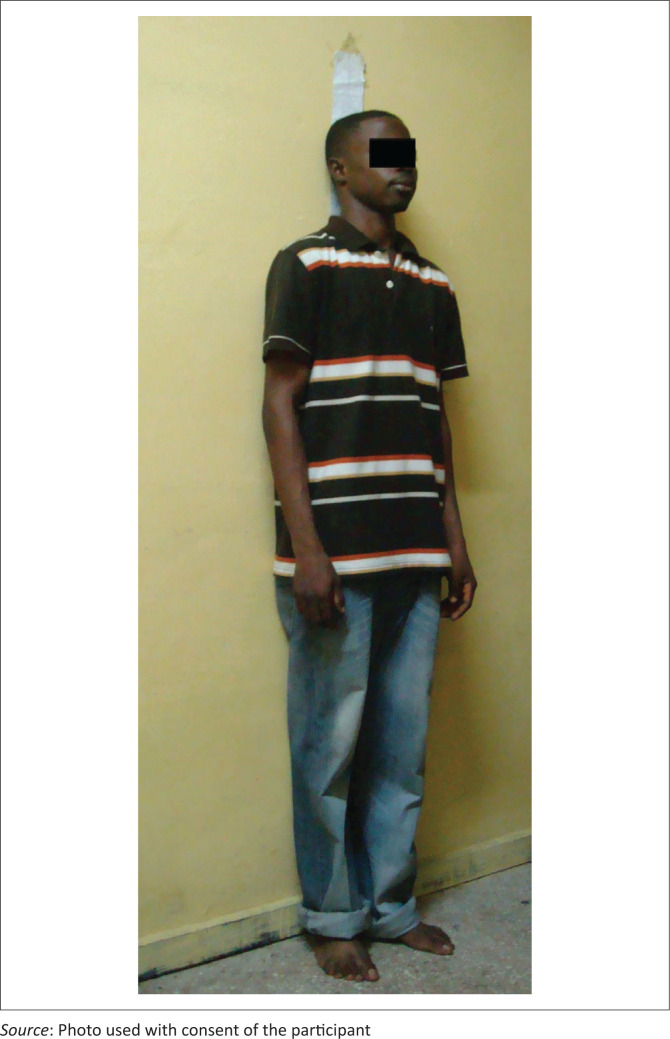
Measurement of height.

**FIGURE 3:**
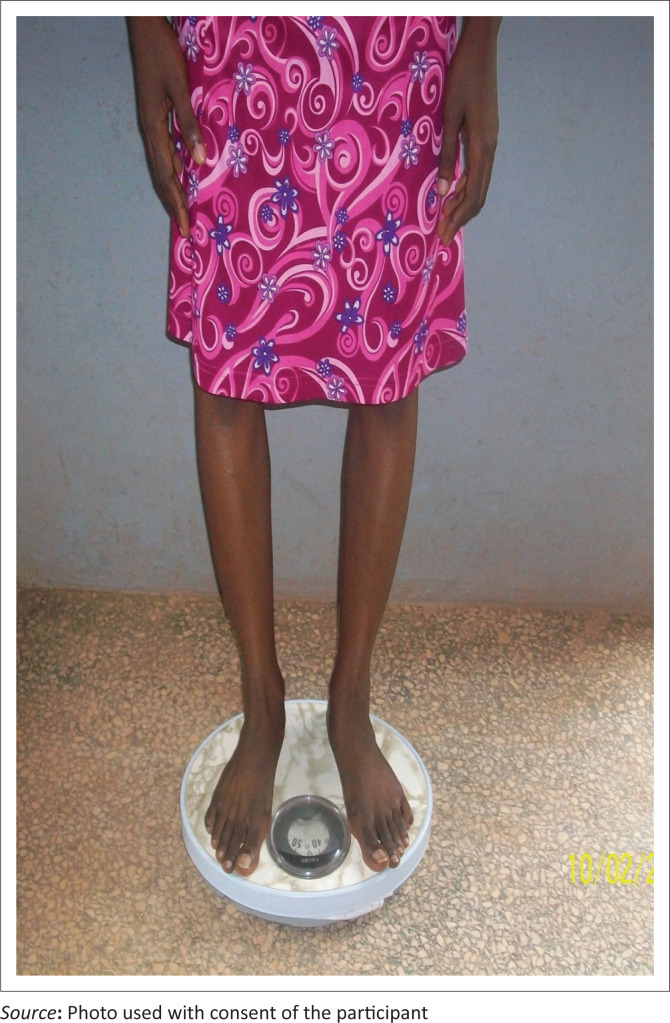
Measurement of weight.

**FIGURE 4:**
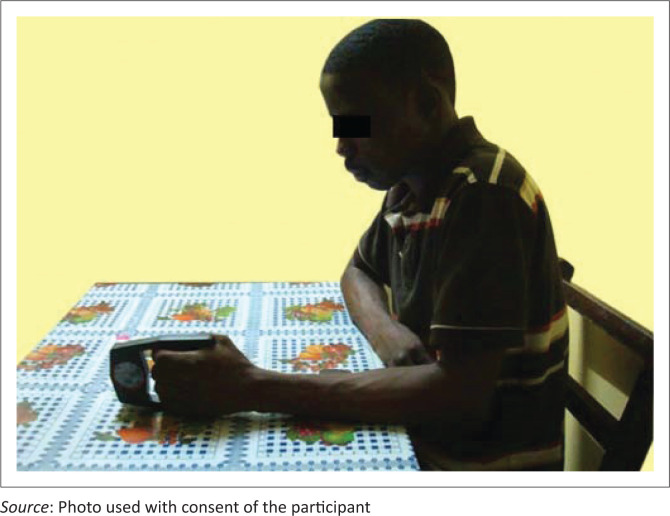
Measurement of hand grip strength.

Students were grouped (left-handed males, right-handed males, left-handed females, and right-handed females) with respect to their dominant hand after the procedure had been explained to them. The dominant hand was determined by asking participants to write anything about their programme of study on a sheet of paper with ruled lines that was provided by us. This was further confirmed by asking them which of their hands was dominant. The height (Figure 2) and weight (Figure 3) of participants were then measured to the nearest 0.1 m and 1.0 kg, respectively. To measure HGS, each participant sat at a table with the shoulder adducted, elbow at 90° of flexion and the forearm and wrist in a neutral position (Figure 4). Participants were then instructed to press the dynamometer as hard as they could. The reading was taken on the third instance. Participants performed three maximum attempts and the average value was recorded. Hand grip measurements were performed for both dominant and non-dominant hands. One-minute rests were given following the initial three attempts to minimise fatigue effects. No verbal encouragements were given to participants as reported by Incel et al. (2002) to ensure that the grip strengths recorded were participants’ maximum voluntary efforts, and to prevent influencing the results obtained. It took about 5 weeks (June 2019 to July 2019) to complete measurements for all the recruited participants.

### Data analysis

Data were analysed using the Statistical Package for the Social Sciences (SPSS) version 24. The mean, frequency distribution and standard deviation (s.d.) were used to summarise all data obtained. Comparison of variables based on gender and dominance were carried out using paired *t*-tests. Pearson’s product moment correlation coefficient was used to test the relationship between height, weight, and BMI and HGS. Data were accepted as statistically significant at *p* < 0.05.

### Ethical considerations

Ethics approval was sought from the Ethics and Protocol Review Committee of the School of Biomedical and Allied Health Sciences, University of Ghana (SBAHS/AA/PT/022006009/20218-2019, 27 May 2021).

## Results

A total of 304 participants, comprising of 177 (58.2%) males and 127 (41.8%) females were recruited. A total of 276 (90.8%) of the participants were right-hand dominant (RHD) while 28 (9.2%) were left-hand dominant (LHD). The mean weight of participants was 62.86 (± 10.30) kg. The mean height was 1.67 (± 0.09) m and the mean BMI was 22.9 (±4.9) kg/m^2^. The mean grip strengths for the various groups are shown in Table 1. Paired *t*-test among males and females showed a statistically significant difference between the mean grip strengths of dominant and non-dominant hands of both RHD and LHD males and females as depicted in Table 2. Pearson’s product moment correlation coefficient showed a statistically significant relationship between grip strength and height as well as grip strength and BMI. The relationship between grip strength and weight was not statistically significant as shown in Table 3.

**TABLE 1:** Mean grip strengths for dominant and non-dominant hands (*N* = 304).

Variables (kg)	mean ± s.d.	
	Males (*n* = 177)	Females (*n* = 127)
RHD right grip strength	35.62 ± 7.36	24.60 ± 6.42
RHD left grip strength	32.84 ± 7.37	22.12 ± 5.37
LHD right grip strength	29.63 ± 6.83	19.96 ± 4.52
LHD left grip strength	25.12 ± 3.18	8.35 ± 2.02

RHD, right-hand dominant; LHD, left-hand dominant; s.d., standard deviation.

**TABLE 2:** Comparison of hand grip strengths of dominant and non-dominant hands among males and females (*N* = 304).

Gender	Dominant hand	*n*	Grip strength	mean ± s.d. (kg)	*t*-value	*p*
Males	Right	160	Right	35.62 ± 7.36	7.48	< 0.001*
	Left	-	Left	32.84 ± 7.37	-	-
	Right	17	Right	29.63 ± 6.83	5.14	< 0.001*
	Left	-	Left	25.12 ± 3.18	-	-
Females	Right	116	Right	24.60 ± 6.42	8.02	< 0.001*
	Left	-	Left	22.12 ± 5.37	-	-
	Right	11	Right	19.96 ± 4.52	3.22	0.009*
	Left	-	Left	8.35 ± 2.02	-	-

s.d., standard deviation.

* , significant.

**TABLE 3:** Relationship between hand grip strength and weight, height and body mass index (*N* = 304).

Variables	mean ± s.d.	*r*-value	*t*-value	*p*
Hand grip strength	29.59 ± 8.33 (kg)	-	-	-
Weight	62.86 ± 10.30 (kg)	0.001	0.013	0.982
Hand grip strength	25.90 ± 8.33 (kg)	-	-	-
Height	167.0 ± 9.1 (m)	0.0492	9.826	< 0.001*
Hand grip strength	25.59 ± 8.33 (kg)	-	-	-
Body mass index	22.90 ± 4.90 (kg/m^2^)	0.290	5.276	< 0.001*

s.d., standard deviation.

* , significant.

## Discussion

Most participants of our study were males or RHD which corroborates with studies conducted in Europe and Asia (Bardo et al. 2021; Nikodelis et al. 2021; Rostamzadeh, Saremi & Tabatabaei 2019). There was a limited number (between 1.4% and 9.2%) of LHD participants as shown by most studies conducted in the United Kingdom, Iran, and Greece, respectively (Bardo et al. 2021; Nikodelis et al. 2021; Rostamzadeh et al. 2019). Studies have consistently reported a higher percentage of individuals being RHD as compared with LHD. This pattern is believed to be influenced by both genetic and environmental factors, and is a common finding across different populations (Brandler & Paracchini 2014). Although slightly different, the recorded HGS values among participants, for males (25.12 ± 3.18 – 35.62 ± 7.6) and females (8.35 ± 2.02 – 24.60 ± 6.42), are well within the range of HGS normative values reported for healthy individuals in countries in other regions such as Asia and Europe (McQuiddy et al. 2015; Pratt et al. 2021).

Hand grip strength was 4.0% and 6.0% higher in the dominant hand than the non-dominant hand in RHD males and females, respectively. These findings are similar to the results reported by Wang et al. (2019), in which they report a similar trend (overall grip strength on the dominant side was 5.0% – 5.6% greater than the non-dominant side). In the LHD group of participants, HGS of the dominant hand was 8.0% higher than the non-dominant hand among the male participants. This seems to contradict the general rule that the dominant hand is approximately 10.0% stronger than the non-dominant hand (Hepping et al. 2021; Mahoney et al. 2022). However, our results corroborated the outcome of Wang et al. (2018) who report that the 10.0% rule could not be applied to the whole population as the rule is only valid for right-handed people. It is worth observing that among the female participants who were LHD, the HGS of their dominant hand was approximately 58.0% higher than that of the non-dominant hand. These variations in findings may be attributed to individual differences in hand dominance, differences in data collecting instrument and the specific population.

The relationship between grip strength and anthropometric factors such as weight, height, and BMI was also explored. The results revealed a significant positive correlation between grip strength and both height and BMI, indicating that taller individuals and those with higher BMI values seemed to have greater grip strength (Ploegmakers et al. 2013). We aimed to investigate the relationship between BMI and HGS, whereas Norman et al. (2011) highlight the potential influence of body composition on muscle strength; however, our findings did not show a significant correlation between weight and HGS. This contradicts the results reported by Trivic et al. (2020), who found a strong positive relationship between weight and HGS. The differences between the outcomes of Trivic et al.’s (2020) study and ours could be the differences in sample sizes and testing environments.

However, our study showed a low (*r* = 290) relationship between BMI and HGS. This finding corroborates the findings of Hardy et al. (2013) and Krakauer and Krakauer (2020) who showed a positive correlation between BMI and HGS. Our findings may be attributed to the population’s normal BMI values, indicating good body composition and overall health (Bi et al. 2019). As HGS is a reliable indicator of total body muscle strength (Wind et al. 2010), a healthy population is expected to exhibit higher grip strength levels. It could also be possible that higher BMI values produce greater muscle mass (Pierce et al. 2017). These contribute to increased grip strength, and higher BMI values which are likely to be associated with greater physical activity levels, which could lead to increased muscle and grip strength (Legrand et al. 2013; Roberts et al. 2011). It is important to note that while our study and other studies (Hardy et al. 2013; Krakauer & Krakauer 2020) have shown a positive relationship between BMI and HGS, other factors such as age, gender, and overall health status may also play a role in muscle strength and HGS. It is therefore important to consider these factors when assessing HGS as a clinical tool.

We showed a positive moderate correlation (*r* = 490) between height and HGS. This finding corroborates those by Liao (2016) and Bhattacharjya, & Goswami (2022). This could be attributed to longer arms because of height, and hence, a greater lever arm for generating force (Ploegmakers et al. 2013). This allows for a more efficient use of force, resulting in a higher level of HGS as reported by Liao (2016).

The convenience sampling method used may have introduced selection bias, as it relied on participants who are readily available and willing to participate, potentially limiting the generalisability of the findings to the broader population.

Another limitation was the method used to measure HGS. This setup provided external support that could potentially influence the HGS measurements. Further studies with a larger sample size could be conducted among the Ghanaian population to find the influence of other anthropometric factors on HGS. Establishing normative values for HGS in healthy individuals in Ghana can facilitate accurate assessments of grip strength deficits and the design of personalised rehabilitation programmes. These findings underscore the importance of considering regional factors in grip strength assessment.

## Conclusion

Our study showed that HGS is influenced by hand dominance, gender, height and BMI but not weight. Based on these findings, it is recommended that the client’s dominant hand, gender height and BMI should be taken into consideration during the assessment of HGS for rehabilitation by physiotherapists and other rehabilitation professionals.
